# The economic impact of open lower limb fractures in the Netherlands: *a cost-of-illness study*

**DOI:** 10.1007/s00068-024-02637-1

**Published:** 2024-08-26

**Authors:** M. P. Noorlander-Borgdorff, W. Kievit, G. F. Giannakópoulos, M. Botman, T. N. Tromp, K. Oflazoglu, H. A. Rakhorst, T. de Jong

**Affiliations:** 1 Department of Plastic, Reconstructive, and Hand Surgery, Amsterdam University Medical Center, Amsterdam, The Netherlands; 2 Department of Plastic and Reconstructive Surgery, Radboud University Medical Center, Nijmegen, The Netherlands; 3 Department for Health Evidence, Radboud University Medical Center, IQ Healthcare, Nijmegen, The Netherlands; 4 Trauma Unit, Department of Surgery, Amsterdam University Medical Center, Amsterdam, The Netherlands; 5 Department of Trauma Surgery, Radboud University Medical Center, Nijmegen, The Netherlands; 6 Department of Plastic, Reconstructive and Hand Surgery, Medisch Spectrum Twente, ZGT Almelo, Enschede, The Netherlands

**Keywords:** Open fractures, Lower limb reconstruction, Healthcare financing, Costs analysis

## Abstract

**Purpose:**

To estimate the one-year sum of direct costs related to open lower limb fracture treatment in an academic setting in the Netherlands. The secondary objective was to estimate the impact of deep infection and nonunion on one-year total direct costs.

**Methods:**

A multi-center, retrospective cost analysis of open lower limb fractures treated in an academic setting in the Netherlands, between 1 January 2017 and 31 December 2018, was conducted. The costing methodology was based on patient level aggregation using a bottom-up approach. A multiple linear regression model was used to predict the total costs based on Fracture-related-infections, multitrauma, intensive care unit (ICU) admission, Gustilo-Anderson grade and nonunion.

**Results:**

Overall, 70 fractures were included for analysis, the majority Gustilo-Anderson grade III fractures (57%). Median (IQR) one-year hospital costs were €31,258 (20,812–58,217). Costs were primarily attributed to the length of hospital stay (58%) and surgical procedures (30%). The median length of stay was 16 days, with an increase to 50 days in Fracture-related infections. Subsequent costs (46,075 [25,891–74,938] vs. 15,244 [8970–30,173]; *p* = 0.002), and total hospital costs (90,862 [52,868–125,004] vs. 29,297 [21,784–40,677]; *p* < 0.001) were significantly higher for infected cases. It was found that Fracture-related infection, multitrauma, and Gustilo-Anderson grade IIIA-C fractures were significant predictors of increased costs.

**Conclusion:**

In treatment of open lower limb fractures, deep infection, higher Gustilo-Anderson classification, and multitrauma significantly increase direct hospital costs. Considering the impact of infection on morbidity and total healthcare costs, future research should focus on preventing Fracture-related infections.

**Supplementary Information:**

The online version contains supplementary material available at 10.1007/s00068-024-02637-1.

## Introduction

Open lower limb fractures are among the most frequent types of open fractures and are often challenging to manage [2–5]. Despite improved treatment options and national guidelines, complications such as deep infection, non-union, and secondary amputation frequently occur [1, 5, 6]. Patients undergo lengthy treatment with substantial impact on the health-related quality of life, the healthcare system, and society [7–9]. Previous studies have shown that open lower limb fractures result in high costs for hospitals and patients [10–12]. Hoekstra et al*.* assessed direct hospital costs in a Belgium hospital following open tibia fractures, reporting length of hospital stay (LOHS) as main cost driver [12]. Severe open tibia fractures, Gustilo-Anderson (GA) Grade III, incurred higher costs attributed to longer total length of stay and prolonged antibiotics use compared to the GA Grade I open tibia fractures [12].

The unsustainable rise in healthcare costs in high income countries had led to a demand for more cost information, transparency, and strategies to improve quality of care associated with lower healthcare costs [7]. Although literature on epidemiology and long-term consequences of traumatic injuries is growing, to our knowledge, detailed information on costs of open lower limb fractures is scarce [10, 13]. Current evidence is derived predominantly from the USA, from small sample sizes, or showing great variance in open fracture severity and anatomical properties [11, 12]. Moreover, little is known about main cost drivers and factors influencing these costs in the Netherlands. Nonetheless, this information is essential for public health policy and development of guidelines in order to improve patient outcome and reduce healthcare costs. Therefore, we aim to explore direct one-year hospital costs in academic setting, main cost drivers, and additional costs related to complications.

## Methods

**Fig. 1:**
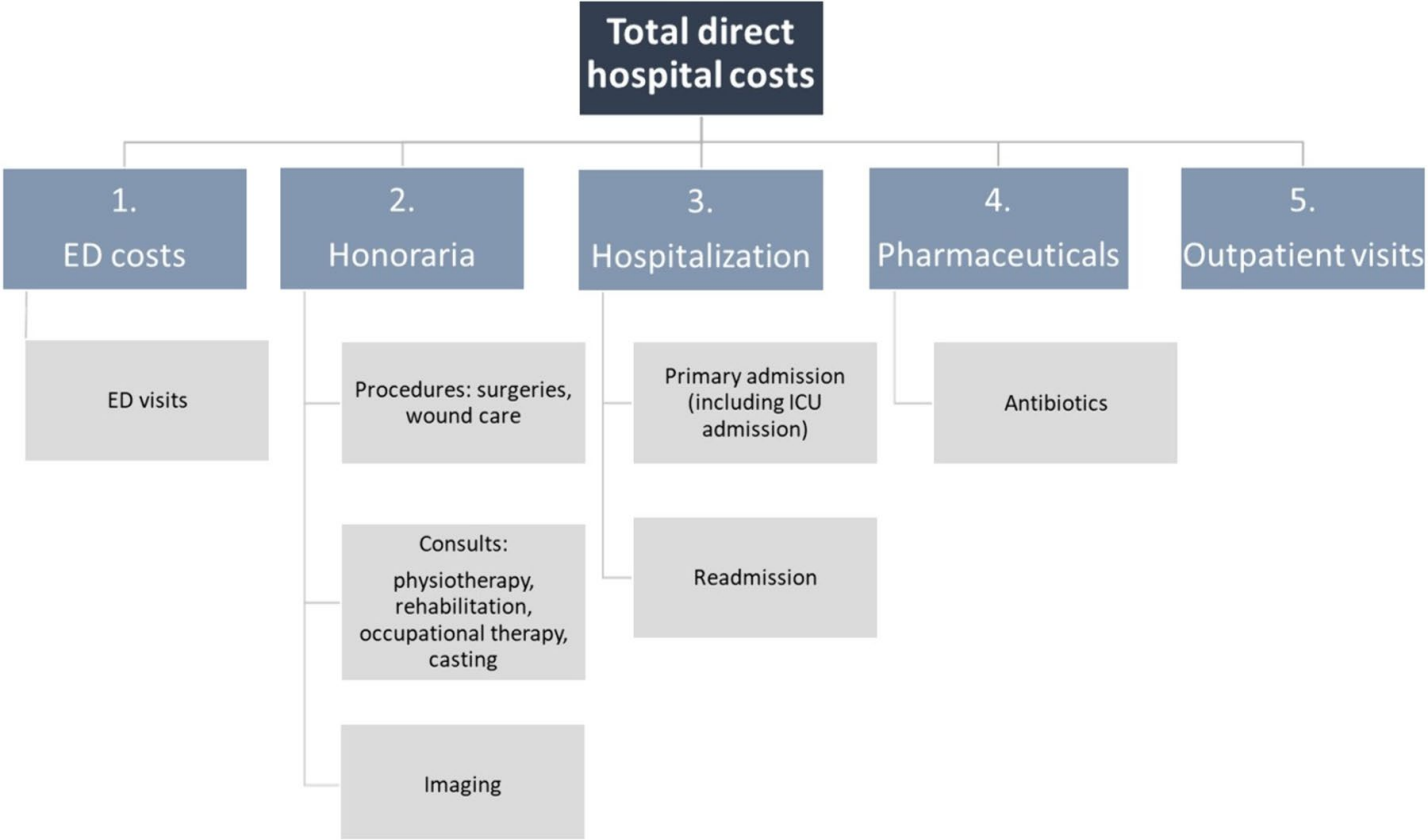
Cost categories of direct hospital costs in open lower limb fracture treatment

This observational cost-of-illness study was performed in continuation of the multicenter International Lower Limb Collaborative study (INTELLECT) in collaboration with the department of IQ Healthcare at the Radboud University [1]. Data from two academic, level 1 trauma centers were used from the INTELLECT database, including open femur, tibia, and hindfoot fractures, GA grade I to IIIC, all patients were treated from injury till one year post-injury [14]. Details of the primary study protocol were previously published by Berner et al. [1]. Clinical INTELLECT data were complemented with hospital registration data on the use of hospital care. This included the following patient data; number of emergency department visits, data on length of stay, Intensive care unit (ICU) admission, readmission, CBV-code^1^ and type of surgical procedures, imaging, consults, emergency department visits, outpatient clinic visits, and antibiotic use (Fig. 1). The Dutch CBV classification system was used for the retrieval of surgical interventions. This is a classification of medical activities within the Dutch Healthcare tariffs organization. Multitrauma was defined as injuries leading to an injury severity score (ISS) of 16 or higher [15]. Nonunion was defined as lack of union requiring surgical interventions after definitive wound closure or incomplete radiographic healing at 1 year [1]. Fracture-related infection (FRI) was defined as a deep infection below muscle fascia, which required surgical exploration for lavage, removal of metalwork, and/or further bone debridement. The study was conducted in compliance with guidelines of the ethics committee of both institutions.

### Costs of hospital care valuation

Direct average hospital-related healthcare costs were calculated using a bottom-up approach (person-based data) from a healthcare providers perspective. Unit costing was performed in adherence to the Dutch manual for costing studies in healthcare [16]. Consumer indexing to June 2023 was applied to the estimated costs in 2014. See data supplement for a detailed overview of unit costs (Table 1). Average or median costs were calculated by multiplication of the individual care units defined as hospital length of stay and readmission (in days), diagnostics (units), emergency department visits (units), outpatient visits (units), surgical procedures (units per individual CBV code), antibiotics (dosage, duration), and consults (units). Four information sources for unit costs were used: iMTA costing tool, average CBV costs for procedures, the Dutch Healthcare Authority tariffs (NZa) and Medicijnkosten.nl [16–18].

**Table 1:** Reference costs

Resource	Unit costs (Euro) 2023*
Attendance of Emergency Department	€ 328,99
*Hospitalization*	
University hospital (per day)	€ 815,48
*Outpatient department visit*	
University hospital (per visit)	€ 207,05
*Diagnostics*	
MRI lower extremity	€ 290,88
CT lower extremity	€ 177,83
Imaging during surgical procedures	€ 184,17
X-ray lower limb	€ 85,35
X-ray femur	€ 47,30
X-ray ankle, hindfoot, foot, toes	€ 47,66
CT-angiography	€ 296,77
Ultrasound lower extremity	€ 85,00
Arterial duplex	€ 149,84
Medical procedures	*CBV cost prices*^*#*^
*Rehabilitation*	
Occupational therapy (per session)	€ 41,92
Physiotherapy (per session)	€ 41,92
Rehabilitation medicine (per consult)	€ 194,34
Pharmaceutical costs	Individual antibiotics (including €6.00 for regular deliveries)

*Costs were adjusted using the consumer pricing index. Reference: central bureau of statistics data (CBS 2023 June):

StatLine—Jaarmutatie consumentenprijsindex; vanaf 1963 (cbs.nl): https://www.cbs.nl/nl-nl/cijfers/detail/70936ned

^#^Average costs for individual CBV codes for specific procedures were obtained from three academic centers:

VU University Medical Center, Academical Medical Centre (AMC), Radboud University Medical Center)

Averages CBV costs are periodically calculated by individual hospitals using the average length of the specific procedures and includes overhead costs (28%). The average costs for the two academic centers were adjusted using the consumer pricing index.

### Statistics

Descriptive analyses were performed in order to summarize the data by cost categories and patient characteristics. Normally distributed data were presented using mean and standard deviation, whereas non-normally distributed data were presented using the median and interquartile range. The Mann–Whitney U test was used to compare differences between two independent groups for nonparametric data. The Kruskal–Wallis test was used when comparing multiple groups. Multiple linear regression analysis was performed including GA grade, multitrauma, intensive care admission, FRI, and non-union as independent variables and total costs as dependent variable. We used bias corrected accelerated bootstrapping to correct for the skewed distribution of the data, to minimize bias and estimate the accuracy of the confidence interval. Backward stepwise regression was performed to reduce multicollinearity. A p-value of smaller than 0.05 was considered significant for all tests. For statistical expediency, we have assumed independence among the 70 fractures, as we do not anticipate that this assumption will impact the outcomes. The statistical analyses were executed through IBM SPSS Statistics 28.0 [19].

## Results

### Study population

In total, 68 patients with 70 fractures were included in this study. The median age (IQR) was 49 (27–61) and the majority was male (69%). The most common cause of injury was road traffic accidents (76%) and the most common type of injury was open tibia fractures (n = 56, 80%), followed by femoral fractures (n = 9, 13%) and fractures of the hindfoot (n = 5, 7%). GA Grade II open fractures were most prevalent (n = 30, 43%) and 17 patients (25%) suffered from GA grade IIIB or IIIC open fractures. Other patient characteristics are shown in Table 2. Two patients were treated for open fractures in both legs. Primary intramedullary nailing was performed in 44%, while 27% underwent external fixation, 24% underwent Open Reduction Internal Fixation (ORIF) and in 4% of patients percutaneous kirschner wire fixation were used for fractures of the hindfoot. FRI occurred in 14% (n = 10) and nonunion in 16% (n = 11). Patients were admitted for a median of 19 days, including readmissions (IQR = 9–35) and underwent a median of three (IQR = 2–6) surgical procedures. Additional clinical data are shown in Table 3.

**Table 2:** Patient characteristics

All open lower limb fractures (N = 70)	N (%)
*Gender*	
Male	48 (69)
Female	22 (31)
*Age category*	
< 18	5 (7)
18–34	19 (27)
35–44	8 (11)
45–54	11 (16)
55–64	14 (20)
65–74	4 (6)
> 75	9 (13)
*Comorbidity*	
Diabetes	6 (9)
Peripheral arterial disease	1 (1)
Hypertension	6 (9)
Asthma	*4 (6)*
COPD	1 (1)
Ischemic heart disease	4 (6)
Cerebrovascular disease	8 (12)
*Gustilo classification*	
Grade 1	10 (14)
Grade 2	30 (43)
Grade 3A	11 (16)
Grade 3B	14 (20)
Grade 3C	3 (4)
Missing	2 (3)
*Etiology*	
Road traffic	53 (76)
Low-energy fall	2 (3)
High energy fall sports	7 (10)
Crush injuries	4 (6)
Sports	1 (1)
Other	3 (4)

**Table 3:** Treatment and clinical outcome data

All open lower limb fractures (N = 70)	N (%)
*Methods of soft tissue reconstruction*	
Conventional dressing	13 (19)
Negative-pressure dressing	18 (26)
Skin graft	18 (74)
Local flap (random pattern)	5 (7)
Regional perforator flap (perforator or axial pattern)	6 (9)
Free flap	6 (9)
*Primary mode of fixation*	
External fixation with rods and pins	16 (23)
External fixation with frame	3 (4)
Plates and screws	17 (24)
Intramedullary nail	31 (44)
Kirschner wires	3 (4)
*Amputation*	
Immediately	2 (3)
Early	2 (3)
Delayed	1 (1)
*Complications*	
Wound infection	15 (21)
FRI	10 (14)
Nonunion	11 (16)
Partial Flap failure	3 (18)
Total flap failure	1 (6)

### Direct hospital costs and complications

Estimated median one-year total direct hospital costs for all patients were €31,258 (IQR 20,812–58,217). In contrast, median [IQR] costs were three-fold higher in the FRI group compared to the uncomplicated group (€90,662 [52,868–125,004] vs. €29,297 [21,784–40,677], *p* < 0.001). In the nonunion group (infected and non-infected) the costs were 52% higher compared to the group without complications, with a median €56,845 [36,655–104,576] vs. €29,297 [21,784–40,677], *p* = 0.05).

A multiple linear regression model with bias corrected accelerated bootstrap was used to predict the total costs based on the following variables: FRI, multitrauma, ICU admission, GA grade, and nonunion. The model had an adjusted R^2^ of 0.45 (F(2, 63) = 19, *p* < 0.001). It was found that FRI, multitrauma and GA grade IIIA or higher were significant predictors of increased total costs. The regression analysis is shown in Supplementary Table 1.

### Cost categories and the role of length of stay

**Fig. 2:**
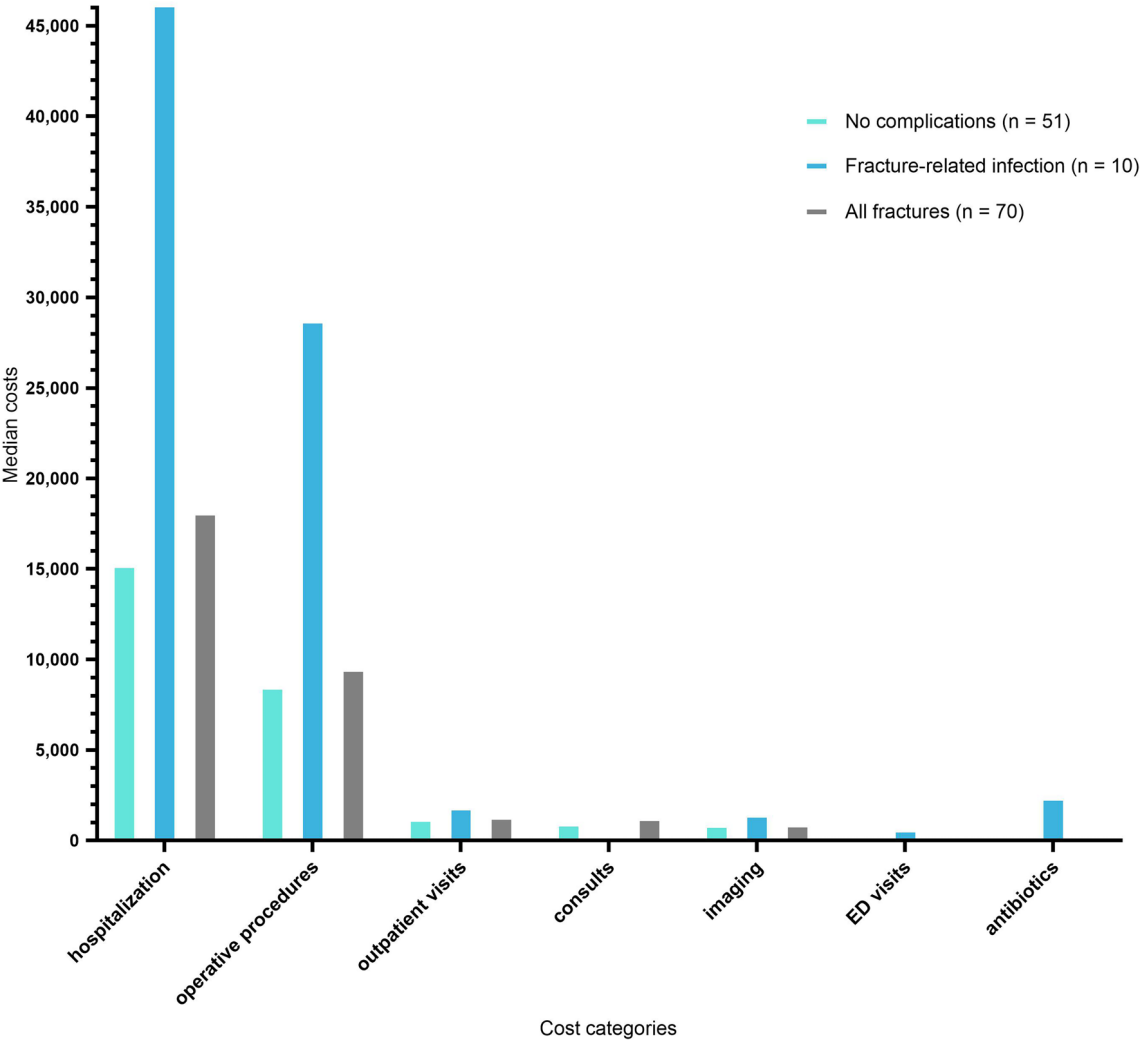
Fracture-related infections (FRI) and the direct one year hospital costs per category

The direct hospital costs in relation to the different cost categories are shown in Fig. 2 and Table 4. Sixty-one percent of costs were attributed to the length of hospitalization, followed by the operative procedures (29.9%) (Table 4). The overall median [IQR] length of stay for the primary admission was 15 days [9–27]. Readmissions led to a median overall one-year length of stay of 19 days [36–9]. FRI patients were admitted longer than uncomplicated cases (median 51 days (IQR 32–61) vs. 16 days (IQR 8–29); Z =  – 3.626, *p* < 0.001), leading to higher admission and total costs (z =  – 3.09, *p* < 0.002; z =  – 3.53, *p* < 0.001) (Table 5).

**Table 4:** Hospital healthcare costs for open lower extremity fractures in euros (N = 70)

Cost category	All patients (*N* = 70) median (IQR)	No complications (*N* = 51)* median (IQR)	FRI (*N* = 10) median (IQR)	Nonunion (*N* = 11) median (IQR)	Total costs (N = 70)	Relative share %
Hospitalization. including readmissions	€18,167 (9,027–35,292)	€15,244 (8,970–30,173)	€46,075 (25,891–74,938)	€33,774 (16,310–71,375)	€2,002,767	61.1
Operative procedures	€9,512 (5,735–17,335)	€8,544 (5,494–12,794)	€ 28,768 (17,811–39,272)	€17,661 (10,435–35,378)	€916,030	27.9
Outpatient visits	€1346 (828–2122)	€1242 (621–1863)	€1864 (1020–3727)	€1863 (1242–3106)	€115,534	3.5
Consults	€1278 (586–1984)	€973 (50–2044)	€1740 (1360–1938)	€1778 (1258–1964)	€110,482	3.4
Imaging	€945 (536–1,527)	€904 (510–1309)	€1464 (1020–2200)	€1528 (1109–2164)	€71,975	2.2
ED visits	€329 (329–658)	€329 (329–329)	€658 (329–740)	€658 (329–987)	€29,938	0.9
Antibiotics	€58 (44–200)	€57 (43–65)	€2415 (383–4353)	€470 (52–3406)	€32,048	1.0
Total costs	€31,258 (20,812–58,217)	€29,297 (21,784–40,677)	€90,862 (52,868–125,004)	€56,845 (36,655–104,576)	€3,278,773	100

Relative share calculated by dividing total costs per category by total costs all patients

*No complications is defined as no deep infection. nonunion or secondary amputation. Deep infection is defined as acute or late deep infection or osteomyelitis below the fascia or bone infection based on clinical

radiographic or microbiological findings within one year follow-up

**Table 5:** Length of stay and number of procedures

Patient groups	All patients (N = 70)	No complications (N = 55)*	FRI (N = 10)	*p*—value ^#^
Length of hospital stay primary admission (in days)	15 (9–27)	14 (8–27)	22 (17–39)	0.042
Total length of hospital stay including readmissions (in days)	19 (9–36)	17 (8–29)	51 (32–61)	< 0.001
Number of procedures	3 (2–6)	3 (2–5)	11 (6–13)	< 0.001

*No complications is defined as no deep infection. nonunion or secondary amputation. Deep infection is defined as acute or late deep infection or osteomyelitis below the fascia or bone infection based on clinical radiographic or microbiological findings within one year follow-up

^H^ Mann–Whitney U Test

### Characteristics of high-costs patients

Patients with GA IIIB and C fractures underwent more procedures, with prolonged total LOHS, leading to higher overall costs compared to Grade IIIA, II and I (Table 6). Overall, patients with GA grade IIIA-C (40% of the cohort) contributed to 55.2% of the total costs. The subgroup GA grade IIIB-C (24.3% if the sample) contributed to 42.9% of the total direct hospital costs, patients with grade II (42.9% of the sample) contributed to 36.8% of the total costs.

**Table 6:** Hospital healthcare costs associated with Gustilo-Anderson grade classification

Gustilo-Anderson grade	I (N = 10)	II (N = 30)	IIIA (N = 11)	IIIB-IIIC (N = 17)	*p*-value*
*Volume*					
Number of operative procedures	2 (1–2)	3 (2–6)	10 (4–13)	10 (5–13)	< 0.001
Length of total hospital stay (in days)	11 (3–30)	23 (4–32)	16 (10–21)	30 (18–62)	< 0.001
*Costs*					
Hospitalization, including readmissions (median, IQR)	€ 8970 (2446–24,057)	€ 18,348 (8036–26,299)	€ 13,920 (9,027–18,393)	€ 46,482 (24,113–80,959)	< 0.001
Operative procedures (median, IQR)	€ 6998 (4325–9,810)	€ 8394 (5374–11,697)	€ 10,715 (5494–12,223)	€ 20,673 (8650–36,292)	0.004
Outpatient clinic visits (median, IQR)	€ 931 (621–1656)	€ 1346 (828–2950)	€ 1242 (828–3002)	€ 1656 (467–2588)	0.546
Consults (median, IQR)	€ 528 (232–684)	€ 1228 (668–2147)	€ 1324 (603–1861)	€ 1861 (1174–3463)	< 0.001
Imaging (median, IQR)	€ 661 (467–855)	€ 985 (393–1476)	€ 919 (803–1251)	€ 1607 (574–1753)	0.016
ED visits (median, IQR)	€ 329 (329–411)	€ 329 (329–411)	€ 329 (329–329)	€ 329 (329–658)	0.520
Antibiotics (median, IQR)	€ 25 (22–61)	€ 58 (53–76)	€ 52 (43–211)	€ 559 (59–2720)	0.002
Total costs (median, IQR)	€ 24,466 (10,258–34,164)	€ 29,487 (18,598–51,050)	€ 29,046 (23,170–39,634)	€ 76,558 (40,736–112,470)	< 0.001

*Kruskall-Wallis Test

The five patients with the highest total costs (7% of the sample) were responsible for 20% of all costs (range €116,382–€158,318). All five patients suffered from GA grade IIIB or IIIC fractures. Three were admitted to the ICU due to concomitant head injuries, three suffered a FRI, leading to nonunion (n = 2) or amputation (n = 1), and one had multiple comorbidities.

## Discussion

In this multicenter study we have estimated the cost-of-illness of open lower limb fractures in the Netherlands. We evaluated the one-year direct hospital costs and reported a median total hospital cost of €31,258 in an academic level 1 trauma center setting. Factors associated with higher healthcare costs were FRI, multitrauma and severity of the injury (GA grade IIIA or higher). FRI led to a three-fold increase in the overall direct one-year cost to a median of €90,662. Costs were primarily attributed to the length of hospital stay, followed by surgical procedures.

Severely, and or multitrauma patients are at a higher risk of infection due to the extent of soft tissue damage, contaminated wounds and the immunological response to trauma. High injury severity scores are associated with an increase in emergency interventions and complications rates [20]. In this study we found that FRI’s were associated with an increase in healthcare use and consequent costs, however, interestingly, ICU admission was not a predictive or confounding factor for higher healthcare costs. Therefore, it is possible that the extent of the injuries associated with ICU admission, for example in concomitant neurotrauma, are of less of a financial impact compared to the extent of the soft tissue injuries of the tibia/fibula and the risk of FRI.

Similar to previous research on the direct costs of open lower limb fractures from countries such as the USA, Canada, Denmark, and Belgium, we found that these injuries are associated with significant healthcare utilization and consequent costs [11, 21]. Schade et al. (2021) reported in a systematic review on open tibia fractures (n = 17,073) that the duration of stay and total costs varied widely, even within countries. The costs in pounds converted to euros ranged from £5,705 to £126,479 (€6,680–€148,157) in high income countries, with the highest costs in the USA population. The median costs for open tibia fracture treatment in this study corresponds to the one year costs for a kidney transplant (36,036–38,666) [22]. Variation in costs in the present study may partly be attributed to the varying severity levels and nature of the injuries, as some patients with concomitant injuries were admitted for a longer period. Nonetheless, it was deemed inappropriate to compare these specific costs between countries as the individual healthcare systems, the methods for data collection, and the financial valuation vary substantially.

Schade et al*.* reported a substantially higher mean length of hospital stay of 56 days compared to our study, but included studies published before and after 2000, some of which were primarily focused on complex reconstruction and amputations. Two other recent studies reported a median length of stay of 10–12 days during the stay for definitive treatment, excluding readmissions [12, 23]. In line with previous findings, we found that hospital length of stay was the main driver of total hospital costs. It was previously reported that infection and nonunion increase the hospital costs and length of stay [11, 24]. Hoekstra et al*.* reported a fivefold increase in costs in patients with deep infections after two years follow-up [12]. It is possible that the total costs in this current study would have further increased, as late complications, such as low-grade FRI, associated non-unions, and osteomyelitis can occur after the 12-month period. Moreover, the question remains if adherence to the guidelines, such as timely treatment and prompt soft tissue coverage, is cost-effective and effective in preventing (infectious) complications. These are important issues for future research. Moreover, FRI is associated with prolonged periods of decreased quality of life (QoL) and substantial absenteeism, potentially adding 3–50% to the total health care expenditure [25–27]. Indirect costs, such as absenteeism from paid and unpaid work as well as decreased productivity, lead to further increases in costs for society.

Strategies to prevent complications and reduce healthcare costs in open lower limb fractures should focus on the following three pillars; (1) developing practice standards (2) prevention of (deep) infections, and (3) early detection and treatment of FRI. The first pillar is supported by the findings of our recent systematic review which showed that direct admission to a specialized center reduces the likelihood of both overall and deep infections, possibly decreasing the LOHS and total costs [28]. Examples include developing practice standards in terms of infrastructure and agreements on rapid fix and flap by means of early plastic surgery consults in case of severe open fractures, available microsurgical services and multidisciplinary teams with scheduled day time joint OR time. Furthermore, methods of prevention such as timely soft tissue coverage, prophylactic (local) antibiotics are strategies that have been shown to lead to reduced infection rates [14, 28–30]. Lastly, the consensus definition of FRI, published in 2018 facilitates a diagnostic algorithm, enabling earlier diagnosis and treatment according to the practice standards as defined in the Dutch FRI guideline [31]. Future research should aim to further improve these strategies and include focus on the rehabilitation process and socio-economic impact.

Inherent to the retrospective nature of this study there are limitations. Our study focused on patients treated primarily in an academic hospital setting, therefore it is possible that the injuries had a higher complexity compared to the general hospitals, with consequent higher costs. Furthermore, productivity losses in terms of absenteeism are part of the total societal costs as stated above, but we were unable to collect these data due to the retrospective nature of this study [25–27]. However, as we approached the economic burden from a healthcare provider perspective, taking into account the Dutch healthcare reimbursement system and guidelines, we estimated the total overall healthcare costs in relation to this perspective, shedding light on the importance of infection prevention. Moreover, inherent to this type of study, an important limitation is that these findings are applicable specifically to the Dutch Healthcare system; differences in reimbursement and healthcare systems limit the possibility of extrapolating to other countries.

Future research involving a larger population with more extensive follow-up is required to evaluate treatment options and their impact on total healthcare-related expenditures, particularly in the context of preventing and promptly treating FRI. This research should include a prospective follow-up of patients spanning both academic and non-academic settings, and account for out-of-pocket expenses and indirect costs, such as productivity losses. By including these economic factors, we hope to assess the overall economic burden and evaluate strategies to further improve patient outcomes. Specifically, it should consider the costs associated with adhering to treatment guidelines, such as prompt soft tissue coverage, to determine effective approaches for enhancing outcomes and reducing costs.

## Conclusion

In conclusion, we report the estimate of the total direct hospital costs of complicated and uncomplicated open lower limb fractures within the Dutch healthcare system. Key factors contributing to the total healthcare costs were FRI, multitrauma, and higher GA classification. Concluding that total healthcare cost is primarily driven by length of hospital stay. Future research with a larger population and more extensive follow-up is needed to evaluate treatment options and their impact on healthcare costs for FRI.

## Supplementary Information

Below is the link to the electronic supplementary material.

Supplementary file1 (DOCX 25 KB)

## Data Availability

No datasets were generated or analysed during the current study.

## Footnotes

1 Centraal Beheer Verrichtingenbestand (CBV) = Central Management Transaction File.
