# Supplementary material for: The economic impact of open lower limb fractures in the Netherlands: a cost-of-illness study
Source: Eur J Trauma Emerg Surg. 2024 Aug 26;50(5):2605–13. doi: 10.1007/s00068-024-02637-1 (PMC11599625; doi:10.1007/s00068-024-02637-1)
Supplement: Supplementary file 1 — Supplementary file1 (DOCX 25 KB) [file 68_2024_2637_MOESM1_ESM.docx]

**Supplemental file**

**The economic impact of open lower limb fractures in the Netherlands: *a cost-of-illness study***

M. P. Noorlander-Borgdorff ^1,2^, W. Kievit^3^, G.F. Giannakópoulos^4^, M. Botman^1^, T.N. Tromp^5^, K. Oflazoglu^1^, H. A. Rakhorst^6^, T. de Jong^2^

1. Department of Plastic, Reconstructive, and Hand Surgery, Amsterdam University Medical Center, Amsterdam, the Netherlands.
2. Department of Plastic and Reconstructive Surgery, Radboud University Medical Center, , Nijmegen, Netherlands
3. Radboud University Medical Center, IQ Healthcare, Department for Health Evidence, Nijmegen, The Netherlands.
4. Trauma unit, Department of Surgery, Amsterdam University Medical Center, Amsterdam, the Netherlands.
5. Department of Trauma Surgery, Radboud University Medical Center, Nijmegen, The Netherlands
6. Department of Plastic, Reconstructive and Hand Surgery, Medisch Spectrum Twente, Enschede, ZGT Almelo, the Netherlands

Corresponding author: M.P. Noorlander-Borgdorff

E-mail: [M.borgdorff@amsterdamumc.nl](mailto:M.borgdorff@amsterdamumc.nl)

Supplemental table 1.

| **Labels** | **Adjusted R^2^** | **Regression coefficients** | **95% CI regression coefficients** | | **Standardized regression coefficients** | **P-values** |
| --- | --- | --- | --- | --- | --- | --- |
|  |  |  | Lower | Upper |  |  |
| **Overall** | 0.452 |  |  | |  | 0.001 |
| **Intercept** |  | 21,421 | 15,727 | 27,566 |  |  |
| **Deep infection** |  | 49,912 | 30,795 | 72,747 | 0.481 | <0.001 |
| **GA grade 3A-C** |  | 20,039 | 4,217 | 35,259 | 0.255 | 0.009 |
| **Multitrauma** |  | 31,538 | 18,937 | 43,472 | 0.418 | <0.001 |

1. Following variables were tested and selected if significant (Multitrauma, IC admission, Gustilo grade (I, III, IIIA-C), nonunion)
2. Bootstrap (1000 samples), Bca (bias corrected)
